# Methyl (2*Z*)-2-(2-fluoro-4-meth­oxy­benzyl­idene)-5-(4-meth­oxy­phen­yl)-7-methyl-3-oxo-2,3-dihydro-5*H*-[1,3]thia­zolo[3,2-*a*]pyrimidine-6-carboxyl­ate

**DOI:** 10.1107/S1600536811025141

**Published:** 2011-07-06

**Authors:** Hoong-Kun Fun, Wan-Sin Loh, B. K. Sarojini, K. Umesha, B. Narayana

**Affiliations:** a X-ray Crystallography Unit, School of Physics, Universiti Sains Malaysia, 11800 USM, Penang, Malaysia; b Department of Chemistry, P. A. College of Engineering, Mangalore 574 153, India; c Department of Studies in Chemistry, Mangalore University, Mangalagangotri 574 199, India

## Abstract

The asymmetric unit of the title compound, C_24_H_21_FN_2_O_5_S, consists of two crystallographically independent mol­ecules. In each mol­ecule, the central dihydro­pyrimidine ring is significantly puckered and adopts a conformation which is best described as an inter­mediate between a boat and a screw boat. The least-squares planes of the dihydro­pyrimidine rings are almost coplanar with the fluoro-substituted benzene rings, making dihedral angles of 9.04 (7) and 6.68 (7)°, and almost perpendicular to the meth­oxy-substituted benzene rings with dihedral angles of 89.23 (7) and 88.30 (7)°. In the mol­ecular structure, *S*(6) ring motifs are formed by C—H⋯O and C—H⋯S hydrogen bonds. In the crystal, mol­ecules are linked into a three-dimensional network by inter­molecular C—H⋯O and C—H⋯F hydrogen bonds. The crystal structure is further stabilized by a C—H⋯π inter­action.

## Related literature

For background to pyrimidine and its derivatives, see: Brugnatelli (1818 ▶); Smee *et al.* (1987 ▶); Lagu *et al.* (2000 ▶). For background to thia­zole and its derivatives, see: Holla *et al.* (2003 ▶); Narayana *et al.* (2004 ▶); Sarojini *et al.* (2010 ▶). For the effect of fluorine in a mol­ecule on its biological activity, see: Filler & Kobayashi (1982 ▶). For related structures, see: Fischer *et al.* (2007 ▶); Jotani *et al.* (2010 ▶). For hydrogen-bond motifs, see: Bernstein *et al.* (1995 ▶). For the stability of the temperature controller used in the data collection, see: Cosier & Glazer (1986 ▶). For puckering parameters, see: Cremer & Pople (1975) ▶.
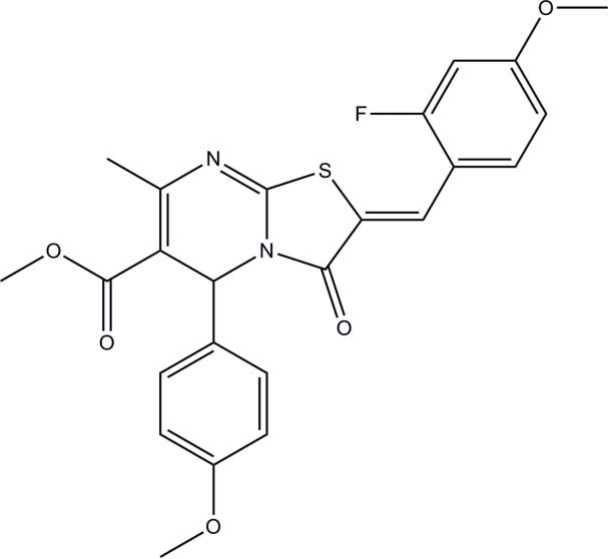


## Experimental

### 

#### Crystal data

C_24_H_21_FN_2_O_5_S
*M* *_r_* = 468.49
Triclinic, 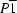

*a* = 11.7374 (2) Å
*b* = 14.3062 (2) Å
*c* = 14.5552 (2) Å
α = 61.939 (1)°
β = 80.791 (1)°
γ = 84.878 (1)°
*V* = 2128.69 (6) Å^3^
*Z* = 4
Mo *K*α radiation
μ = 0.20 mm^−1^
*T* = 100 K
0.37 × 0.33 × 0.10 mm

#### Data collection

Bruker SMART APEXII CCD area-detector diffractometer
Absorption correction: multi-scan (*SADABS*; Bruker, 2009 ▶) *T*_min_ = 0.929, *T*_max_ = 0.980
46547 measured reflections
12629 independent reflections
10022 reflections with *I* > 2σ(*I*)
*R*_int_ = 0.031

#### Refinement

*R*[*F* ^2^ > 2σ(*F* ^2^)] = 0.044
*wR*(*F* ^2^) = 0.123
*S* = 1.04
12629 reflections
602 parameters
H-atom parameters constrained
Δρ_max_ = 0.49 e Å^−3^
Δρ_min_ = −0.49 e Å^−3^

### 

Data collection: *APEX2* (Bruker, 2009 ▶); cell refinement: *SAINT* (Bruker, 2009 ▶); data reduction: *SAINT*; program(s) used to solve structure: *SHELXTL* (Sheldrick, 2008 ▶); program(s) used to refine structure: *SHELXTL*; molecular graphics: *SHELXTL*; software used to prepare material for publication: *SHELXTL* and *PLATON* (Spek, 2009 ▶).

## Supplementary Material

Crystal structure: contains datablock(s) global, I. DOI: 10.1107/S1600536811025141/is2738sup1.cif

Structure factors: contains datablock(s) I. DOI: 10.1107/S1600536811025141/is2738Isup2.hkl

Supplementary material file. DOI: 10.1107/S1600536811025141/is2738Isup3.cml

Additional supplementary materials:  crystallographic information; 3D view; checkCIF report

## Figures and Tables

**Table 1:** Hydrogen-bond geometry (Å, °) *Cg*1 is the centroid of the C17*A*–C22*A* ring.

*D*—H⋯*A*	*D*—H	H⋯*A*	*D*⋯*A*	*D*—H⋯*A*
C12*A*—H12*A*⋯O1*B*^i^	0.95	2.52	3.4472 (18)	166
C13*A*—H13*A*⋯S1*A*	0.95	2.55	3.2321 (18)	129
C13*B*—H13*B*⋯S1*B*	0.95	2.54	3.2578 (19)	133
C14*A*—H14*A*⋯O3*A*	0.98	2.17	2.927 (2)	133
C14*B*—H14*D*⋯O3*B*	0.98	2.15	2.8820 (19)	130
C18*A*—H18*A*⋯O3*B*^ii^	0.95	2.58	3.249 (2)	128
C21*A*—H21*A*⋯F1*B*^iii^	0.95	2.48	3.2047 (18)	134
C24*A*—H24*C*⋯O3*B*	0.98	2.48	3.2724 (19)	137
C24*A*—H24*A*⋯*Cg*1^iv^	0.98	2.50	3.3612 (19)	147

Symmetry codes: (i) 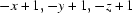; (ii) 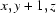; (iii) 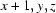; (iv) 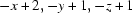.

**Fig. 1.**
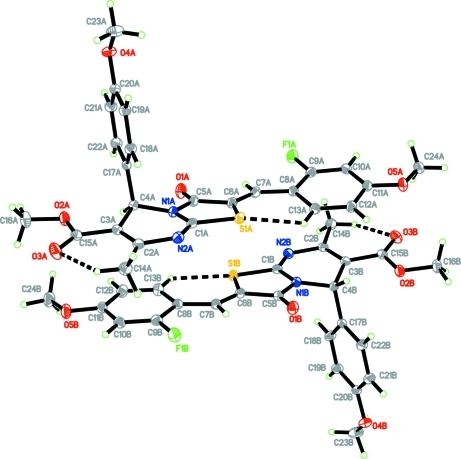
The molecular structure of the title compound, showing 50% probability displacement ellipsoids and the atom-numbering scheme. The dashed line indicates the intramolecular hydrogen bond.

**Fig. 2.**
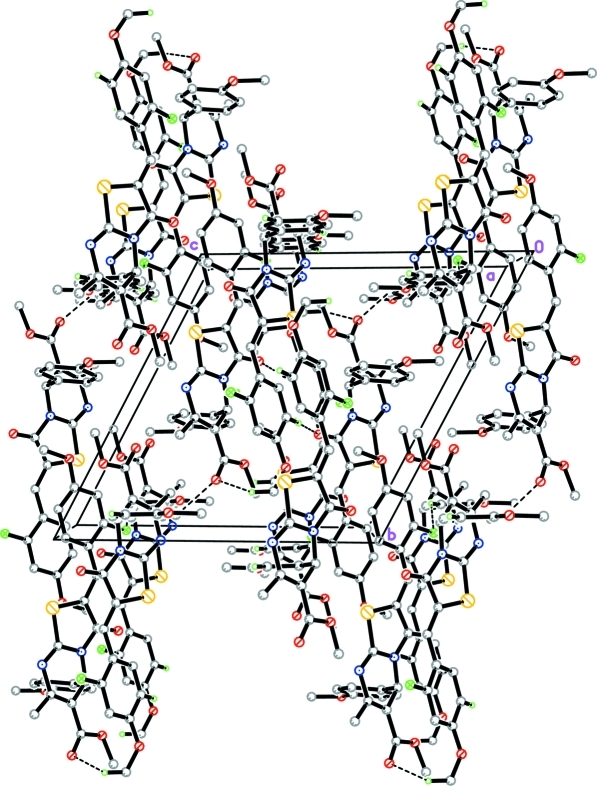
The crystal packing of the title compound, viewed along the a axis. H atoms not involved in the intermolecular interactions (dashed lines) have been omitted for clarity.

## supplementary crystallographic information

### Comment

Pyrimidine derivatives are known for their varied biological properties. Brugnatelli was the first scientist to isolate '*Alloxan*', a pyrimidine derivative in 1818, and later this compound was found to possess antineoplastic properties (Brugnatelli, 1818). Nucleosides of pyrimidine bases have been used extensively as antiviral and anticancer agents (Smee *et al.*, 1987). Recently, fluoropyrimidines and flurouracil-based combination therapy is used in the treatment of gastrointestinal cancer and solid tumors. Furopyrimidinones are found to be metabolites of dihydropyrimidinones that are subtype-selective antagonists of the α_1a_-adreginic receptor antagonists (Lagu *et al.*, 2000). Thiazoles and their derivatives are found to be associated with various biological activities such as antibacterial, antifungal and anti-inflammatory activities (Holla *et al.*, 2003; Narayana *et al.*, 2004; Sarojini *et al.*, 2010). Presence of fluorine in a molecule enhances drug persistence and lipid solubility (Filler & Kobayashi, 1982). The crystal structure of ethyl 7-methyl-2-[4-(methylsulfanyl)benzylidene] -5-[4-(methylsulfanyl)phenyl]-3-oxo-2,3-dihydro-5*H*-thiazolo [3,2-*a*] pyrimidine-6-carboxylate synthesized in an one pot reaction using tin(II) chloride was reported (Fischer *et al.*, 2007). In continuation to our studies on crystal structure of new heterocyclic analogs, we report the synthesis and crystal structure of a new methyl (2*Z*)-2-[(2-fluoro-4-methoxyphenyl)methylidene]-5-(4-methoxyphenyl)-7- methyl-3-oxo-2,3-dihydro-5*H*-[1,3]thiazolo[3,2-*a*]pyrimidine-6- carboxylate, C_24_H_20_N_2_FO_5_S.

The title compound (Fig. 1) consists of two crystallographically independent molecules (molecule *A* & *B*). The central pyrimidine rings (N1/C1/N2/C2–C4) are significantly puckered and adopt a conformation which is best described as an intermediate between a boat and screw boat with the puckering parameter (Cremer & Pople, 1975), Q = 0.2565 (15) Å, Θ = 112.0 (3)° and φ = 136.5 (4)° in molecule *A*; Q = 0.2340 (15) Å, Θ = 69.0 (4)° and φ = 317.1 (4)° in molecule *B*. The mean planes of pyrimidine rings are almost coplanar with the fluoro-substituted phenyl rings (C8–C13) with dihedral angles of 9.04 (7)° in molecule *A* and 6.68 (7)° in molecule *B*; and almost perpendicular with the methoxy-substituted phenyl rings (C17–C22) with dihedral angles of 89.23 (7) and 88.30 (7)°, respectively, in molecule *A* and *B*. In the molecular structure, *S*(6) ring motifs (Bernstein *et al.*, 1995) are formed *via* intramolecular C14A—H14A···O3A, C14B—H14D···O3B, C13A—H13A···S1A and C13B—H13B···S1B hydrogen bonds (Table 1). Bond lengths and angles are within the normal ranges and are comparable to the related structure (Jotani *et al.*, 2010).

In the crystal packing (Fig. 2), the molecules are linked into three-dimensional network by intermolecular C12A—H12A···O1B, C18A—H18A···O3B, C21A—H21A···F1B and C24A—H24C···O3B hydrogen bonds (Table 1). The crystal structure is further stabilized by C—H···π interactions (Table 1), involving the centroids of C17A–C22A ring (*Cg*1).

### Experimental

A mixture of methyl 4-(4-methoxyphenyl)-methyl-2-thioxo-1,2,3,4-tetrahydropyrimidine-5-carboxylate (2.59 mmol), chloro acetic acid (0.29 g, 3.11 mmol), 2-fluoro-4-methoxy-benzaldehyde (3.11 mmol) and sodium acetate (10 mmol) in 1:1 mixture glacial acetic acid/ acetic anhydride (10 ml) were heated to 130 °C for 3–5 h. After the completion of the reaction, the reaction mass was cooled to room temperature and quenched to ice cooled water. The solid precipitated out were collected by filtration and recrystallized from acetonitrile/water to afford title compound (*m.p.* 477.9–478.5 K). Analysis, found (calculated): C 61.50 (61.53), H 4.45 (4.48), N 5.97% (5.98%).

### Refinement

All H atoms were positioned geometrically and refined using a riding model with *U*_iso_(H) = 1.2 or 1.5*U*_eq_(C) (C—H = 0.95–1.00 °). A rotating group model was applied to the methyl groups. There is no pseudo-symmetry in the crystal structure.

### Crystal data

**Table:**

C_24_H_21_FN_2_O_5_S	*Z* = 4
*M**_r_* = 468.49	*F*(000) = 976
Triclinic, *P*1	*D*_x_ = 1.462 Mg m^−^^3^
Hall symbol: -P 1	Mo *K*α radiation, λ = 0.71073 Å
*a* = 11.7374 (2) Å	Cell parameters from 9870 reflections
*b* = 14.3062 (2) Å	θ = 2.5–30.2°
*c* = 14.5552 (2) Å	µ = 0.20 mm^−^^1^
α = 61.939 (1)°	*T* = 100 K
β = 80.791 (1)°	Plate, yellow
γ = 84.878 (1)°	0.37 × 0.33 × 0.10 mm
*V* = 2128.69 (6) Å^3^	

### Data collection

**Table:**

Bruker SMART APEXII CCD area-detector diffractometer	12629 independent reflections
Radiation source: fine-focus sealed tube	10022 reflections with *I* > 2σ(*I*)
graphite	*R*_int_ = 0.031
φ and ω scans	θ_max_ = 30.3°, θ_min_ = 1.6°
Absorption correction: multi-scan (*SADABS*; Bruker, 2009)	*h* = −16→16
*T*_min_ = 0.929, *T*_max_ = 0.980	*k* = −19→20
46547 measured reflections	*l* = −20→20

### Refinement

**Table:**

Refinement on *F*^2^	Primary atom site location: structure-invariant direct methods
Least-squares matrix: full	Secondary atom site location: difference Fourier map
*R*[*F*^2^ > 2σ(*F*^2^)] = 0.044	Hydrogen site location: inferred from neighbouring sites
*wR*(*F*^2^) = 0.123	H-atom parameters constrained
*S* = 1.04	*w* = 1/[σ^2^(*F*_o_^2^) + (0.0608*P*)^2^ + 0.8664*P*] where *P* = (*F*_o_^2^ + 2*F*_c_^2^)/3
12629 reflections	(Δ/σ)_max_ = 0.001
602 parameters	Δρ_max_ = 0.49 e Å^−^^3^
0 restraints	Δρ_min_ = −0.49 e Å^−^^3^

### Special details

**Table:**

Experimental. The crystal was placed in the cold stream of an Oxford Cryosystems Cobra open-flow nitrogen cryostat (Cosier & Glazer, 1986) operating at 100.0 (1) K.
Geometry. All e.s.d.'s (except the e.s.d. in the dihedral angle between two l.s. planes) are estimated using the full covariance matrix. The cell e.s.d.'s are taken into account individually in the estimation of e.s.d.'s in distances, angles and torsion angles; correlations between e.s.d.'s in cell parameters are only used when they are defined by crystal symmetry. An approximate (isotropic) treatment of cell e.s.d.'s is used for estimating e.s.d.'s involving l.s. planes.
Refinement. Refinement of *F*^2^ against ALL reflections. The weighted *R*-factor *wR* and goodness of fit *S* are based on *F*^2^, conventional *R*-factors *R* are based on *F*, with *F* set to zero for negative *F*^2^. The threshold expression of *F*^2^ > σ(*F*^2^) is used only for calculating *R*-factors(gt) *etc*. and is not relevant to the choice of reflections for refinement. *R*-factors based on *F*^2^ are statistically about twice as large as those based on *F*, and *R*- factors based on ALL data will be even larger.

### Fractional atomic coordinates and isotropic or equivalent isotropic displacement parameters (Å^2^)

**Table:**

	*x*	*y*	*z*	*U*_iso_*/*U*_eq_	
S1A	0.74753 (3)	0.78373 (3)	0.38302 (3)	0.01691 (8)	
F1A	1.06880 (7)	0.51889 (7)	0.31316 (7)	0.02532 (19)	
O1A	0.97502 (9)	0.88905 (8)	0.13570 (8)	0.0236 (2)	
O2A	0.86743 (9)	1.26518 (8)	0.02597 (8)	0.0226 (2)	
O3A	0.70722 (10)	1.31812 (9)	0.09628 (9)	0.0289 (2)	
O4A	1.26523 (9)	1.11070 (9)	0.31666 (8)	0.0220 (2)	
O5A	0.83717 (9)	0.25364 (8)	0.60271 (8)	0.0228 (2)	
N1A	0.83373 (10)	0.95336 (9)	0.22110 (9)	0.0154 (2)	
N2A	0.67609 (10)	0.98277 (10)	0.32890 (9)	0.0182 (2)	
C1A	0.74917 (12)	0.92232 (11)	0.30607 (10)	0.0160 (2)	
C2A	0.67868 (12)	1.09231 (11)	0.25526 (11)	0.0171 (3)	
C3A	0.76554 (12)	1.13335 (11)	0.17452 (11)	0.0163 (3)	
C4A	0.86866 (11)	1.06546 (10)	0.16296 (10)	0.0152 (2)	
H4AA	0.8902	1.0849	0.0869	0.018*	
C5A	0.89772 (12)	0.87326 (11)	0.20725 (11)	0.0169 (3)	
C6A	0.85833 (12)	0.76833 (11)	0.29416 (10)	0.0165 (3)	
C7A	0.91184 (12)	0.67857 (11)	0.29940 (11)	0.0171 (3)	
H7AA	0.9724	0.6883	0.2441	0.021*	
C8A	0.88975 (12)	0.56950 (11)	0.37761 (10)	0.0164 (3)	
C9A	0.97110 (12)	0.49082 (11)	0.38290 (11)	0.0175 (3)	
C10A	0.95972 (12)	0.38516 (11)	0.45515 (11)	0.0183 (3)	
H10A	1.0185	0.3348	0.4558	0.022*	
C11A	0.85951 (12)	0.35454 (11)	0.52711 (11)	0.0177 (3)	
C12A	0.77288 (12)	0.43003 (11)	0.52394 (11)	0.0182 (3)	
H12A	0.7036	0.4090	0.5719	0.022*	
C13A	0.78905 (12)	0.53461 (11)	0.45091 (11)	0.0173 (3)	
H13A	0.7302	0.5851	0.4499	0.021*	
C14A	0.57542 (12)	1.15044 (12)	0.27907 (12)	0.0227 (3)	
H14A	0.5819	1.2262	0.2299	0.034*	
H14B	0.5713	1.1399	0.3511	0.034*	
H14C	0.5054	1.1233	0.2718	0.034*	
C15A	0.77248 (12)	1.24770 (11)	0.09754 (11)	0.0179 (3)	
C16A	0.88851 (16)	1.37461 (12)	−0.04928 (13)	0.0290 (3)	
H16A	0.9594	1.3794	−0.0970	0.044*	
H16B	0.8971	1.4152	−0.0122	0.044*	
H16C	0.8234	1.4036	−0.0897	0.044*	
C17A	0.97262 (11)	1.08027 (10)	0.20475 (10)	0.0146 (2)	
C18A	0.96020 (12)	1.08372 (11)	0.29973 (11)	0.0168 (3)	
H18A	0.8853	1.0790	0.3377	0.020*	
C19A	1.05591 (12)	1.09396 (11)	0.34035 (11)	0.0177 (3)	
H19A	1.0462	1.0959	0.4055	0.021*	
C20A	1.16530 (12)	1.10132 (11)	0.28444 (11)	0.0173 (3)	
C21A	1.17872 (12)	1.09913 (12)	0.18825 (11)	0.0198 (3)	
H21A	1.2534	1.1047	0.1498	0.024*	
C22A	1.08299 (12)	1.08886 (11)	0.14903 (11)	0.0181 (3)	
H22A	1.0926	1.0877	0.0835	0.022*	
C23A	1.25509 (14)	1.10826 (15)	0.41683 (13)	0.0281 (3)	
H23A	1.3318	1.1143	0.4318	0.042*	
H23B	1.2207	1.0413	0.4712	0.042*	
H23C	1.2058	1.1675	0.4162	0.042*	
C24A	0.91703 (14)	0.17216 (12)	0.60228 (12)	0.0240 (3)	
H24A	0.8921	0.1039	0.6615	0.036*	
H24B	0.9940	0.1880	0.6087	0.036*	
H24C	0.9196	0.1687	0.5363	0.036*	
S1B	0.73247 (3)	0.73597 (3)	0.09732 (3)	0.01691 (8)	
F1B	0.43727 (7)	1.00519 (7)	0.19386 (7)	0.02408 (19)	
O1B	0.50193 (9)	0.63422 (8)	0.34200 (8)	0.0209 (2)	
O2B	0.61941 (9)	0.25875 (8)	0.46823 (8)	0.0207 (2)	
O3B	0.78696 (9)	0.20771 (8)	0.40519 (8)	0.0236 (2)	
O4B	0.21975 (9)	0.37224 (9)	0.19460 (8)	0.0222 (2)	
O5B	0.63291 (10)	1.27590 (8)	−0.10488 (8)	0.0250 (2)	
N1B	0.64551 (10)	0.56854 (9)	0.26041 (9)	0.0151 (2)	
N2B	0.81022 (10)	0.53686 (9)	0.16058 (9)	0.0173 (2)	
C1B	0.73321 (11)	0.59824 (11)	0.17804 (10)	0.0157 (2)	
C2B	0.80789 (11)	0.42898 (11)	0.23771 (10)	0.0160 (2)	
C3B	0.71905 (11)	0.38889 (11)	0.31656 (10)	0.0151 (2)	
C4B	0.61373 (11)	0.45619 (10)	0.32381 (10)	0.0147 (2)	
H4BA	0.5924	0.4411	0.3987	0.018*	
C5B	0.57923 (11)	0.64961 (11)	0.27055 (10)	0.0160 (2)	
C6B	0.61960 (11)	0.75356 (11)	0.18334 (10)	0.0161 (2)	
C7B	0.56982 (12)	0.84384 (11)	0.17922 (10)	0.0164 (3)	
H7BA	0.5093	0.8343	0.2345	0.020*	
C8B	0.59422 (12)	0.95327 (11)	0.10361 (10)	0.0162 (3)	
C9B	0.52370 (12)	1.03373 (11)	0.11276 (11)	0.0176 (3)	
C10B	0.53657 (12)	1.13969 (11)	0.04417 (11)	0.0194 (3)	
H10B	0.4859	1.1910	0.0535	0.023*	
C11B	0.62607 (13)	1.17023 (11)	−0.03983 (11)	0.0189 (3)	
C12B	0.70110 (13)	1.09380 (12)	−0.05141 (11)	0.0214 (3)	
H12B	0.7630	1.1142	−0.1074	0.026*	
C13B	0.68413 (13)	0.98811 (12)	0.01961 (11)	0.0202 (3)	
H13B	0.7357	0.9368	0.0111	0.024*	
C14B	0.91448 (12)	0.37015 (12)	0.22093 (12)	0.0206 (3)	
H14D	0.9023	0.2938	0.2633	0.031*	
H14E	0.9796	0.3904	0.2420	0.031*	
H14F	0.9314	0.3878	0.1465	0.031*	
C15B	0.71571 (12)	0.27668 (11)	0.39846 (10)	0.0166 (3)	
C16B	0.60587 (14)	0.15225 (12)	0.55346 (12)	0.0261 (3)	
H16D	0.5295	0.1458	0.5944	0.039*	
H16E	0.6656	0.1369	0.5989	0.039*	
H16F	0.6134	0.1019	0.5249	0.039*	
C17B	0.51005 (11)	0.43441 (10)	0.28660 (10)	0.0150 (2)	
C18B	0.51751 (12)	0.44252 (12)	0.18702 (11)	0.0193 (3)	
H18B	0.5886	0.4618	0.1415	0.023*	
C19B	0.42220 (12)	0.42283 (12)	0.15258 (11)	0.0202 (3)	
H19B	0.4282	0.4292	0.0840	0.024*	
C20B	0.31845 (12)	0.39380 (11)	0.21960 (11)	0.0173 (3)	
C21B	0.31040 (12)	0.38513 (12)	0.32011 (11)	0.0204 (3)	
H21B	0.2396	0.3655	0.3659	0.025*	
C22B	0.40572 (12)	0.40517 (12)	0.35291 (11)	0.0187 (3)	
H22B	0.3998	0.3989	0.4214	0.022*	
C23B	0.22887 (14)	0.37021 (14)	0.09641 (13)	0.0274 (3)	
H23D	0.1544	0.3509	0.0883	0.041*	
H23E	0.2880	0.3179	0.0947	0.041*	
H23F	0.2505	0.4404	0.0389	0.041*	
C24B	0.71782 (15)	1.30983 (13)	−0.19621 (12)	0.0299 (3)	
H24D	0.7114	1.3868	−0.2390	0.045*	
H24E	0.7051	1.2748	−0.2375	0.045*	
H24F	0.7950	1.2912	−0.1746	0.045*	

### Atomic displacement parameters (Å^2^)

**Table:**

	*U*^11^	*U*^22^	*U*^33^	*U*^12^	*U*^13^	*U*^23^
S1A	0.01787 (16)	0.01556 (16)	0.01531 (15)	−0.00280 (12)	0.00011 (12)	−0.00582 (12)
F1A	0.0188 (4)	0.0223 (4)	0.0273 (5)	−0.0011 (3)	0.0040 (3)	−0.0074 (4)
O1A	0.0270 (5)	0.0179 (5)	0.0206 (5)	−0.0025 (4)	0.0056 (4)	−0.0069 (4)
O2A	0.0240 (5)	0.0152 (5)	0.0208 (5)	−0.0008 (4)	0.0003 (4)	−0.0029 (4)
O3A	0.0318 (6)	0.0199 (5)	0.0287 (6)	0.0079 (5)	−0.0022 (5)	−0.0080 (5)
O4A	0.0152 (5)	0.0293 (6)	0.0234 (5)	−0.0021 (4)	−0.0046 (4)	−0.0130 (5)
O5A	0.0272 (5)	0.0144 (5)	0.0213 (5)	−0.0013 (4)	−0.0024 (4)	−0.0039 (4)
N1A	0.0160 (5)	0.0137 (5)	0.0146 (5)	−0.0019 (4)	−0.0014 (4)	−0.0050 (4)
N2A	0.0163 (5)	0.0179 (6)	0.0197 (6)	−0.0016 (4)	−0.0016 (4)	−0.0082 (5)
C1A	0.0153 (6)	0.0177 (6)	0.0145 (6)	−0.0031 (5)	−0.0027 (5)	−0.0064 (5)
C2A	0.0146 (6)	0.0185 (6)	0.0197 (6)	0.0003 (5)	−0.0040 (5)	−0.0096 (5)
C3A	0.0150 (6)	0.0149 (6)	0.0181 (6)	0.0010 (5)	−0.0048 (5)	−0.0064 (5)
C4A	0.0153 (6)	0.0141 (6)	0.0153 (6)	−0.0012 (5)	−0.0028 (5)	−0.0057 (5)
C5A	0.0187 (6)	0.0152 (6)	0.0166 (6)	−0.0017 (5)	−0.0022 (5)	−0.0069 (5)
C6A	0.0177 (6)	0.0169 (6)	0.0141 (6)	−0.0036 (5)	−0.0020 (5)	−0.0059 (5)
C7A	0.0177 (6)	0.0180 (6)	0.0156 (6)	−0.0032 (5)	−0.0015 (5)	−0.0076 (5)
C8A	0.0180 (6)	0.0156 (6)	0.0162 (6)	−0.0021 (5)	−0.0031 (5)	−0.0074 (5)
C9A	0.0157 (6)	0.0192 (7)	0.0165 (6)	−0.0024 (5)	−0.0013 (5)	−0.0074 (5)
C10A	0.0178 (6)	0.0173 (7)	0.0199 (6)	0.0008 (5)	−0.0050 (5)	−0.0081 (5)
C11A	0.0221 (7)	0.0153 (6)	0.0156 (6)	−0.0022 (5)	−0.0054 (5)	−0.0059 (5)
C12A	0.0188 (6)	0.0185 (7)	0.0159 (6)	−0.0033 (5)	−0.0001 (5)	−0.0071 (5)
C13A	0.0184 (6)	0.0160 (6)	0.0185 (6)	0.0002 (5)	−0.0029 (5)	−0.0089 (5)
C14A	0.0172 (7)	0.0217 (7)	0.0290 (7)	0.0012 (5)	−0.0004 (6)	−0.0126 (6)
C15A	0.0191 (6)	0.0181 (6)	0.0170 (6)	−0.0003 (5)	−0.0060 (5)	−0.0072 (5)
C16A	0.0381 (9)	0.0171 (7)	0.0230 (7)	−0.0027 (6)	−0.0011 (7)	−0.0026 (6)
C17A	0.0137 (6)	0.0119 (6)	0.0159 (6)	0.0000 (5)	−0.0023 (5)	−0.0044 (5)
C18A	0.0139 (6)	0.0182 (6)	0.0187 (6)	−0.0001 (5)	−0.0003 (5)	−0.0093 (5)
C19A	0.0178 (6)	0.0185 (6)	0.0184 (6)	0.0003 (5)	−0.0027 (5)	−0.0099 (5)
C20A	0.0152 (6)	0.0156 (6)	0.0198 (6)	−0.0002 (5)	−0.0046 (5)	−0.0064 (5)
C21A	0.0144 (6)	0.0227 (7)	0.0196 (6)	−0.0012 (5)	−0.0004 (5)	−0.0080 (6)
C22A	0.0167 (6)	0.0200 (7)	0.0155 (6)	−0.0008 (5)	−0.0004 (5)	−0.0069 (5)
C23A	0.0233 (7)	0.0404 (9)	0.0285 (8)	−0.0028 (7)	−0.0067 (6)	−0.0211 (7)
C24A	0.0280 (8)	0.0154 (7)	0.0258 (7)	0.0013 (6)	−0.0098 (6)	−0.0056 (6)
S1B	0.01764 (16)	0.01579 (16)	0.01431 (15)	−0.00132 (12)	0.00060 (12)	−0.00518 (12)
F1B	0.0216 (4)	0.0228 (5)	0.0226 (4)	0.0024 (3)	0.0026 (3)	−0.0085 (4)
O1B	0.0198 (5)	0.0194 (5)	0.0198 (5)	−0.0009 (4)	0.0035 (4)	−0.0079 (4)
O2B	0.0187 (5)	0.0172 (5)	0.0186 (5)	0.0001 (4)	−0.0003 (4)	−0.0027 (4)
O3B	0.0252 (5)	0.0203 (5)	0.0223 (5)	0.0070 (4)	−0.0052 (4)	−0.0080 (4)
O4B	0.0151 (5)	0.0271 (6)	0.0270 (5)	−0.0020 (4)	−0.0050 (4)	−0.0139 (5)
O5B	0.0336 (6)	0.0162 (5)	0.0198 (5)	−0.0040 (4)	−0.0040 (4)	−0.0033 (4)
N1B	0.0144 (5)	0.0144 (5)	0.0146 (5)	−0.0011 (4)	−0.0009 (4)	−0.0054 (4)
N2B	0.0156 (5)	0.0175 (6)	0.0162 (5)	−0.0001 (4)	−0.0004 (4)	−0.0064 (5)
C1B	0.0144 (6)	0.0184 (6)	0.0140 (6)	−0.0021 (5)	−0.0018 (5)	−0.0070 (5)
C2B	0.0143 (6)	0.0174 (6)	0.0174 (6)	0.0004 (5)	−0.0037 (5)	−0.0088 (5)
C3B	0.0140 (6)	0.0158 (6)	0.0157 (6)	0.0013 (5)	−0.0039 (5)	−0.0071 (5)
C4B	0.0135 (6)	0.0141 (6)	0.0140 (6)	−0.0008 (5)	−0.0008 (5)	−0.0047 (5)
C5B	0.0153 (6)	0.0165 (6)	0.0162 (6)	−0.0005 (5)	−0.0031 (5)	−0.0073 (5)
C6B	0.0153 (6)	0.0173 (6)	0.0142 (6)	−0.0019 (5)	−0.0018 (5)	−0.0059 (5)
C7B	0.0153 (6)	0.0181 (6)	0.0152 (6)	−0.0014 (5)	−0.0019 (5)	−0.0071 (5)
C8B	0.0172 (6)	0.0162 (6)	0.0160 (6)	0.0000 (5)	−0.0047 (5)	−0.0073 (5)
C9B	0.0171 (6)	0.0205 (7)	0.0150 (6)	−0.0013 (5)	−0.0028 (5)	−0.0076 (5)
C10B	0.0211 (7)	0.0186 (7)	0.0200 (6)	0.0019 (5)	−0.0068 (5)	−0.0093 (5)
C11B	0.0241 (7)	0.0151 (6)	0.0171 (6)	−0.0031 (5)	−0.0066 (5)	−0.0056 (5)
C12B	0.0236 (7)	0.0208 (7)	0.0176 (6)	−0.0046 (6)	0.0006 (5)	−0.0074 (6)
C13B	0.0219 (7)	0.0185 (7)	0.0198 (6)	−0.0012 (5)	−0.0008 (5)	−0.0090 (6)
C14B	0.0166 (6)	0.0204 (7)	0.0238 (7)	0.0016 (5)	0.0002 (5)	−0.0105 (6)
C15B	0.0173 (6)	0.0182 (6)	0.0152 (6)	−0.0001 (5)	−0.0044 (5)	−0.0078 (5)
C16B	0.0261 (8)	0.0194 (7)	0.0212 (7)	−0.0025 (6)	−0.0013 (6)	−0.0002 (6)
C17B	0.0135 (6)	0.0137 (6)	0.0155 (6)	0.0006 (5)	−0.0024 (5)	−0.0050 (5)
C18B	0.0151 (6)	0.0240 (7)	0.0176 (6)	−0.0035 (5)	0.0002 (5)	−0.0088 (6)
C19B	0.0187 (7)	0.0244 (7)	0.0181 (6)	−0.0013 (5)	−0.0034 (5)	−0.0098 (6)
C20B	0.0144 (6)	0.0149 (6)	0.0222 (6)	0.0007 (5)	−0.0050 (5)	−0.0078 (5)
C21B	0.0146 (6)	0.0233 (7)	0.0216 (7)	−0.0025 (5)	0.0003 (5)	−0.0094 (6)
C22B	0.0161 (6)	0.0216 (7)	0.0165 (6)	−0.0011 (5)	0.0000 (5)	−0.0079 (5)
C23B	0.0211 (7)	0.0368 (9)	0.0344 (8)	−0.0011 (6)	−0.0071 (6)	−0.0238 (8)
C24B	0.0373 (9)	0.0222 (8)	0.0219 (7)	−0.0080 (7)	−0.0019 (7)	−0.0028 (6)

### Geometric parameters (Å, °)

**Table:**

S1A—C6A	1.7581 (14)	S1B—C1B	1.7565 (14)
S1A—C1A	1.7597 (14)	S1B—C6B	1.7585 (14)
F1A—C9A	1.3538 (15)	F1B—C9B	1.3515 (16)
O1A—C5A	1.2131 (17)	O1B—C5B	1.2131 (16)
O2A—C15A	1.3501 (17)	O2B—C15B	1.3469 (16)
O2A—C16A	1.4425 (18)	O2B—C16B	1.4449 (17)
O3A—C15A	1.2048 (18)	O3B—C15B	1.2126 (17)
O4A—C20A	1.3700 (16)	O4B—C20B	1.3689 (16)
O4A—C23A	1.4277 (18)	O4B—C23B	1.4297 (18)
O5A—C11A	1.3580 (16)	O5B—C11B	1.3560 (17)
O5A—C24A	1.4308 (18)	O5B—C24B	1.4322 (19)
N1A—C1A	1.3715 (17)	N1B—C1B	1.3715 (17)
N1A—C5A	1.3903 (18)	N1B—C5B	1.3912 (17)
N1A—C4A	1.4784 (17)	N1B—C4B	1.4749 (17)
N2A—C1A	1.2809 (18)	N2B—C1B	1.2836 (18)
N2A—C2A	1.4224 (18)	N2B—C2B	1.4180 (17)
C2A—C3A	1.3563 (19)	C2B—C3B	1.3557 (18)
C2A—C14A	1.4958 (19)	C2B—C14B	1.4969 (19)
C3A—C15A	1.4860 (19)	C3B—C15B	1.4817 (19)
C3A—C4A	1.5216 (19)	C3B—C4B	1.5191 (18)
C4A—C17A	1.5195 (18)	C4B—C17B	1.5195 (18)
C4A—H4AA	1.0000	C4B—H4BA	1.0000
C5A—C6A	1.4892 (19)	C5B—C6B	1.4848 (19)
C6A—C7A	1.352 (2)	C6B—C7B	1.3479 (19)
C7A—C8A	1.4507 (19)	C7B—C8B	1.4499 (19)
C7A—H7AA	0.9500	C7B—H7BA	0.9500
C8A—C9A	1.391 (2)	C8B—C13B	1.4017 (19)
C8A—C13A	1.4089 (19)	C8B—C9B	1.4023 (19)
C9A—C10A	1.3800 (19)	C9B—C10B	1.3733 (19)
C10A—C11A	1.3922 (19)	C10B—C11B	1.399 (2)
C10A—H10A	0.9500	C10B—H10B	0.9500
C11A—C12A	1.405 (2)	C11B—C12B	1.398 (2)
C12A—C13A	1.3763 (19)	C12B—C13B	1.383 (2)
C12A—H12A	0.9500	C12B—H12B	0.9500
C13A—H13A	0.9500	C13B—H13B	0.9500
C14A—H14A	0.9800	C14B—H14D	0.9800
C14A—H14B	0.9800	C14B—H14E	0.9800
C14A—H14C	0.9800	C14B—H14F	0.9800
C16A—H16A	0.9800	C16B—H16D	0.9800
C16A—H16B	0.9800	C16B—H16E	0.9800
C16A—H16C	0.9800	C16B—H16F	0.9800
C17A—C18A	1.3905 (18)	C17B—C18B	1.3874 (18)
C17A—C22A	1.3963 (18)	C17B—C22B	1.3927 (18)
C18A—C19A	1.3987 (18)	C18B—C19B	1.3968 (19)
C18A—H18A	0.9500	C18B—H18B	0.9500
C19A—C20A	1.3895 (19)	C19B—C20B	1.3914 (19)
C19A—H19A	0.9500	C19B—H19B	0.9500
C20A—C21A	1.3985 (19)	C20B—C21B	1.3970 (19)
C21A—C22A	1.3870 (19)	C21B—C22B	1.3853 (19)
C21A—H21A	0.9500	C21B—H21B	0.9500
C22A—H22A	0.9500	C22B—H22B	0.9500
C23A—H23A	0.9800	C23B—H23D	0.9800
C23A—H23B	0.9800	C23B—H23E	0.9800
C23A—H23C	0.9800	C23B—H23F	0.9800
C24A—H24A	0.9800	C24B—H24D	0.9800
C24A—H24B	0.9800	C24B—H24E	0.9800
C24A—H24C	0.9800	C24B—H24F	0.9800
			
C6A—S1A—C1A	91.13 (6)	C1B—S1B—C6B	91.33 (6)
C15A—O2A—C16A	115.45 (12)	C15B—O2B—C16B	116.22 (11)
C20A—O4A—C23A	116.84 (11)	C20B—O4B—C23B	116.59 (11)
C11A—O5A—C24A	117.68 (12)	C11B—O5B—C24B	116.97 (12)
C1A—N1A—C5A	116.74 (11)	C1B—N1B—C5B	116.75 (11)
C1A—N1A—C4A	119.51 (11)	C1B—N1B—C4B	120.30 (11)
C5A—N1A—C4A	122.50 (11)	C5B—N1B—C4B	122.30 (11)
C1A—N2A—C2A	116.50 (12)	C1B—N2B—C2B	116.32 (12)
N2A—C1A—N1A	126.57 (13)	N2B—C1B—N1B	126.41 (13)
N2A—C1A—S1A	121.71 (11)	N2B—C1B—S1B	122.14 (10)
N1A—C1A—S1A	111.69 (10)	N1B—C1B—S1B	111.42 (10)
C3A—C2A—N2A	121.64 (12)	C3B—C2B—N2B	122.00 (12)
C3A—C2A—C14A	127.04 (13)	C3B—C2B—C14B	126.45 (13)
N2A—C2A—C14A	111.32 (12)	N2B—C2B—C14B	111.53 (12)
C2A—C3A—C15A	123.13 (13)	C2B—C3B—C15B	122.75 (12)
C2A—C3A—C4A	121.29 (12)	C2B—C3B—C4B	121.57 (12)
C15A—C3A—C4A	115.37 (12)	C15B—C3B—C4B	115.64 (11)
N1A—C4A—C17A	109.69 (10)	N1B—C4B—C3B	108.06 (10)
N1A—C4A—C3A	108.04 (11)	N1B—C4B—C17B	110.01 (10)
C17A—C4A—C3A	112.26 (11)	C3B—C4B—C17B	113.02 (11)
N1A—C4A—H4AA	108.9	N1B—C4B—H4BA	108.6
C17A—C4A—H4AA	108.9	C3B—C4B—H4BA	108.6
C3A—C4A—H4AA	108.9	C17B—C4B—H4BA	108.6
O1A—C5A—N1A	123.89 (13)	O1B—C5B—N1B	123.30 (13)
O1A—C5A—C6A	126.57 (13)	O1B—C5B—C6B	127.04 (13)
N1A—C5A—C6A	109.53 (11)	N1B—C5B—C6B	109.65 (11)
C7A—C6A—C5A	119.98 (12)	C7B—C6B—C5B	119.92 (12)
C7A—C6A—S1A	129.08 (11)	C7B—C6B—S1B	129.49 (11)
C5A—C6A—S1A	110.82 (10)	C5B—C6B—S1B	110.59 (10)
C6A—C7A—C8A	129.12 (13)	C6B—C7B—C8B	130.12 (13)
C6A—C7A—H7AA	115.4	C6B—C7B—H7BA	114.9
C8A—C7A—H7AA	115.4	C8B—C7B—H7BA	114.9
C9A—C8A—C13A	115.48 (12)	C13B—C8B—C9B	115.22 (13)
C9A—C8A—C7A	119.74 (12)	C13B—C8B—C7B	125.77 (13)
C13A—C8A—C7A	124.78 (13)	C9B—C8B—C7B	119.02 (12)
F1A—C9A—C10A	117.14 (12)	F1B—C9B—C10B	117.93 (12)
F1A—C9A—C8A	118.38 (12)	F1B—C9B—C8B	117.95 (12)
C10A—C9A—C8A	124.49 (13)	C10B—C9B—C8B	124.12 (13)
C9A—C10A—C11A	118.03 (13)	C9B—C10B—C11B	118.45 (13)
C9A—C10A—H10A	121.0	C9B—C10B—H10B	120.8
C11A—C10A—H10A	121.0	C11B—C10B—H10B	120.8
O5A—C11A—C10A	124.32 (13)	O5B—C11B—C12B	124.75 (13)
O5A—C11A—C12A	115.59 (12)	O5B—C11B—C10B	115.22 (13)
C10A—C11A—C12A	120.09 (13)	C12B—C11B—C10B	120.03 (13)
C13A—C12A—C11A	119.53 (13)	C13B—C12B—C11B	119.23 (13)
C13A—C12A—H12A	120.2	C13B—C12B—H12B	120.4
C11A—C12A—H12A	120.2	C11B—C12B—H12B	120.4
C12A—C13A—C8A	122.35 (13)	C12B—C13B—C8B	122.92 (14)
C12A—C13A—H13A	118.8	C12B—C13B—H13B	118.5
C8A—C13A—H13A	118.8	C8B—C13B—H13B	118.5
C2A—C14A—H14A	109.5	C2B—C14B—H14D	109.5
C2A—C14A—H14B	109.5	C2B—C14B—H14E	109.5
H14A—C14A—H14B	109.5	H14D—C14B—H14E	109.5
C2A—C14A—H14C	109.5	C2B—C14B—H14F	109.5
H14A—C14A—H14C	109.5	H14D—C14B—H14F	109.5
H14B—C14A—H14C	109.5	H14E—C14B—H14F	109.5
O3A—C15A—O2A	122.37 (13)	O3B—C15B—O2B	122.50 (13)
O3A—C15A—C3A	127.66 (14)	O3B—C15B—C3B	127.07 (13)
O2A—C15A—C3A	109.96 (12)	O2B—C15B—C3B	110.43 (11)
O2A—C16A—H16A	109.5	O2B—C16B—H16D	109.5
O2A—C16A—H16B	109.5	O2B—C16B—H16E	109.5
H16A—C16A—H16B	109.5	H16D—C16B—H16E	109.5
O2A—C16A—H16C	109.5	O2B—C16B—H16F	109.5
H16A—C16A—H16C	109.5	H16D—C16B—H16F	109.5
H16B—C16A—H16C	109.5	H16E—C16B—H16F	109.5
C18A—C17A—C22A	118.73 (12)	C18B—C17B—C22B	118.95 (12)
C18A—C17A—C4A	120.87 (12)	C18B—C17B—C4B	120.97 (12)
C22A—C17A—C4A	120.39 (12)	C22B—C17B—C4B	120.08 (12)
C17A—C18A—C19A	121.20 (12)	C17B—C18B—C19B	121.05 (13)
C17A—C18A—H18A	119.4	C17B—C18B—H18B	119.5
C19A—C18A—H18A	119.4	C19B—C18B—H18B	119.5
C20A—C19A—C18A	119.35 (12)	C20B—C19B—C18B	119.40 (13)
C20A—C19A—H19A	120.3	C20B—C19B—H19B	120.3
C18A—C19A—H19A	120.3	C18B—C19B—H19B	120.3
O4A—C20A—C19A	124.58 (12)	O4B—C20B—C19B	124.54 (13)
O4A—C20A—C21A	115.46 (12)	O4B—C20B—C21B	115.59 (12)
C19A—C20A—C21A	119.96 (12)	C19B—C20B—C21B	119.87 (12)
C22A—C21A—C20A	120.02 (13)	C22B—C21B—C20B	119.97 (13)
C22A—C21A—H21A	120.0	C22B—C21B—H21B	120.0
C20A—C21A—H21A	120.0	C20B—C21B—H21B	120.0
C21A—C22A—C17A	120.72 (13)	C21B—C22B—C17B	120.76 (13)
C21A—C22A—H22A	119.6	C21B—C22B—H22B	119.6
C17A—C22A—H22A	119.6	C17B—C22B—H22B	119.6
O4A—C23A—H23A	109.5	O4B—C23B—H23D	109.5
O4A—C23A—H23B	109.5	O4B—C23B—H23E	109.5
H23A—C23A—H23B	109.5	H23D—C23B—H23E	109.5
O4A—C23A—H23C	109.5	O4B—C23B—H23F	109.5
H23A—C23A—H23C	109.5	H23D—C23B—H23F	109.5
H23B—C23A—H23C	109.5	H23E—C23B—H23F	109.5
O5A—C24A—H24A	109.5	O5B—C24B—H24D	109.5
O5A—C24A—H24B	109.5	O5B—C24B—H24E	109.5
H24A—C24A—H24B	109.5	H24D—C24B—H24E	109.5
O5A—C24A—H24C	109.5	O5B—C24B—H24F	109.5
H24A—C24A—H24C	109.5	H24D—C24B—H24F	109.5
H24B—C24A—H24C	109.5	H24E—C24B—H24F	109.5
			
C2A—N2A—C1A—N1A	4.0 (2)	C2B—N2B—C1B—N1B	−4.0 (2)
C2A—N2A—C1A—S1A	−173.94 (9)	C2B—N2B—C1B—S1B	173.68 (9)
C5A—N1A—C1A—N2A	−175.05 (13)	C5B—N1B—C1B—N2B	173.17 (13)
C4A—N1A—C1A—N2A	17.4 (2)	C4B—N1B—C1B—N2B	−15.8 (2)
C5A—N1A—C1A—S1A	3.11 (15)	C5B—N1B—C1B—S1B	−4.76 (14)
C4A—N1A—C1A—S1A	−164.46 (9)	C4B—N1B—C1B—S1B	166.23 (9)
C6A—S1A—C1A—N2A	175.33 (12)	C6B—S1B—C1B—N2B	−173.17 (12)
C6A—S1A—C1A—N1A	−2.93 (10)	C6B—S1B—C1B—N1B	4.85 (10)
C1A—N2A—C2A—C3A	−10.65 (19)	C1B—N2B—C2B—C3B	10.35 (19)
C1A—N2A—C2A—C14A	168.43 (12)	C1B—N2B—C2B—C14B	−168.29 (12)
N2A—C2A—C3A—C15A	−178.59 (12)	N2B—C2B—C3B—C15B	−179.35 (11)
C14A—C2A—C3A—C15A	2.5 (2)	C14B—C2B—C3B—C15B	−0.9 (2)
N2A—C2A—C3A—C4A	−4.1 (2)	N2B—C2B—C3B—C4B	3.03 (19)
C14A—C2A—C3A—C4A	177.00 (13)	C14B—C2B—C3B—C4B	−178.55 (12)
C1A—N1A—C4A—C17A	94.47 (14)	C1B—N1B—C4B—C3B	25.68 (15)
C5A—N1A—C4A—C17A	−72.35 (15)	C5B—N1B—C4B—C3B	−163.84 (11)
C1A—N1A—C4A—C3A	−28.20 (15)	C1B—N1B—C4B—C17B	−98.13 (13)
C5A—N1A—C4A—C3A	164.98 (11)	C5B—N1B—C4B—C17B	72.35 (15)
C2A—C3A—C4A—N1A	22.18 (16)	C2B—C3B—C4B—N1B	−19.80 (16)
C15A—C3A—C4A—N1A	−162.90 (11)	C15B—C3B—C4B—N1B	162.42 (10)
C2A—C3A—C4A—C17A	−98.90 (15)	C2B—C3B—C4B—C17B	102.17 (14)
C15A—C3A—C4A—C17A	76.02 (14)	C15B—C3B—C4B—C17B	−75.61 (14)
C1A—N1A—C5A—O1A	180.00 (13)	C1B—N1B—C5B—O1B	−177.26 (12)
C4A—N1A—C5A—O1A	−12.8 (2)	C4B—N1B—C5B—O1B	11.9 (2)
C1A—N1A—C5A—C6A	−1.46 (16)	C1B—N1B—C5B—C6B	1.79 (16)
C4A—N1A—C5A—C6A	165.70 (11)	C4B—N1B—C5B—C6B	−169.01 (11)
O1A—C5A—C6A—C7A	1.3 (2)	O1B—C5B—C6B—C7B	1.1 (2)
N1A—C5A—C6A—C7A	−177.23 (12)	N1B—C5B—C6B—C7B	−177.93 (12)
O1A—C5A—C6A—S1A	177.66 (12)	O1B—C5B—C6B—S1B	−179.03 (12)
N1A—C5A—C6A—S1A	−0.83 (14)	N1B—C5B—C6B—S1B	1.97 (13)
C1A—S1A—C6A—C7A	178.09 (13)	C1B—S1B—C6B—C7B	176.05 (13)
C1A—S1A—C6A—C5A	2.11 (10)	C1B—S1B—C6B—C5B	−3.83 (10)
C5A—C6A—C7A—C8A	178.41 (12)	C5B—C6B—C7B—C8B	179.49 (12)
S1A—C6A—C7A—C8A	2.7 (2)	S1B—C6B—C7B—C8B	−0.4 (2)
C6A—C7A—C8A—C9A	−164.72 (14)	C6B—C7B—C8B—C13B	−4.7 (2)
C6A—C7A—C8A—C13A	15.9 (2)	C6B—C7B—C8B—C9B	175.42 (14)
C13A—C8A—C9A—F1A	178.69 (11)	C13B—C8B—C9B—F1B	−178.35 (11)
C7A—C8A—C9A—F1A	−0.78 (19)	C7B—C8B—C9B—F1B	1.54 (18)
C13A—C8A—C9A—C10A	−1.7 (2)	C13B—C8B—C9B—C10B	2.0 (2)
C7A—C8A—C9A—C10A	178.86 (12)	C7B—C8B—C9B—C10B	−178.11 (12)
F1A—C9A—C10A—C11A	−179.58 (11)	F1B—C9B—C10B—C11B	179.72 (12)
C8A—C9A—C10A—C11A	0.8 (2)	C8B—C9B—C10B—C11B	−0.6 (2)
C24A—O5A—C11A—C10A	−5.89 (19)	C24B—O5B—C11B—C12B	5.1 (2)
C24A—O5A—C11A—C12A	173.63 (12)	C24B—O5B—C11B—C10B	−175.45 (12)
C9A—C10A—C11A—O5A	−179.63 (12)	C9B—C10B—C11B—O5B	179.39 (12)
C9A—C10A—C11A—C12A	0.86 (19)	C9B—C10B—C11B—C12B	−1.1 (2)
O5A—C11A—C12A—C13A	178.95 (12)	O5B—C11B—C12B—C13B	−179.19 (13)
C10A—C11A—C12A—C13A	−1.5 (2)	C10B—C11B—C12B—C13B	1.4 (2)
C11A—C12A—C13A—C8A	0.5 (2)	C11B—C12B—C13B—C8B	0.1 (2)
C9A—C8A—C13A—C12A	0.98 (19)	C9B—C8B—C13B—C12B	−1.7 (2)
C7A—C8A—C13A—C12A	−179.59 (12)	C7B—C8B—C13B—C12B	178.40 (13)
C16A—O2A—C15A—O3A	2.8 (2)	C16B—O2B—C15B—O3B	0.88 (19)
C16A—O2A—C15A—C3A	−175.94 (11)	C16B—O2B—C15B—C3B	−179.27 (11)
C2A—C3A—C15A—O3A	1.8 (2)	C2B—C3B—C15B—O3B	−1.6 (2)
C4A—C3A—C15A—O3A	−173.06 (14)	C4B—C3B—C15B—O3B	176.18 (13)
C2A—C3A—C15A—O2A	−179.61 (12)	C2B—C3B—C15B—O2B	178.59 (12)
C4A—C3A—C15A—O2A	5.58 (16)	C4B—C3B—C15B—O2B	−3.66 (15)
N1A—C4A—C17A—C18A	−76.38 (15)	N1B—C4B—C17B—C18B	65.35 (16)
C3A—C4A—C17A—C18A	43.75 (17)	C3B—C4B—C17B—C18B	−55.52 (17)
N1A—C4A—C17A—C22A	102.51 (14)	N1B—C4B—C17B—C22B	−115.06 (14)
C3A—C4A—C17A—C22A	−137.36 (13)	C3B—C4B—C17B—C22B	124.07 (14)
C22A—C17A—C18A—C19A	−1.0 (2)	C22B—C17B—C18B—C19B	0.6 (2)
C4A—C17A—C18A—C19A	177.95 (12)	C4B—C17B—C18B—C19B	−179.81 (13)
C17A—C18A—C19A—C20A	0.3 (2)	C17B—C18B—C19B—C20B	−0.5 (2)
C23A—O4A—C20A—C19A	2.7 (2)	C23B—O4B—C20B—C19B	6.1 (2)
C23A—O4A—C20A—C21A	−177.20 (13)	C23B—O4B—C20B—C21B	−173.95 (13)
C18A—C19A—C20A—O4A	−179.44 (13)	C18B—C19B—C20B—O4B	−179.75 (13)
C18A—C19A—C20A—C21A	0.4 (2)	C18B—C19B—C20B—C21B	0.3 (2)
O4A—C20A—C21A—C22A	179.41 (13)	O4B—C20B—C21B—C22B	179.88 (13)
C19A—C20A—C21A—C22A	−0.5 (2)	C19B—C20B—C21B—C22B	−0.2 (2)
C20A—C21A—C22A—C17A	−0.2 (2)	C20B—C21B—C22B—C17B	0.2 (2)
C18A—C17A—C22A—C21A	0.9 (2)	C18B—C17B—C22B—C21B	−0.5 (2)
C4A—C17A—C22A—C21A	−178.01 (13)	C4B—C17B—C22B—C21B	179.95 (13)

### Hydrogen-bond geometry (Å, °)

**Table:**

*Cg*1 is the centroid of the C17A–C22A ring.

**Table:**

*D*—H···*A*	*D*—H	H···*A*	*D*···*A*	*D*—H···*A*
C12A—H12A···O1B^i^	0.95	2.52	3.4472 (18)	166
C13A—H13A···S1A	0.95	2.55	3.2321 (18)	129
C13B—H13B···S1B	0.95	2.54	3.2578 (19)	133
C14A—H14A···O3A	0.98	2.17	2.927 (2)	133
C14B—H14D···O3B	0.98	2.15	2.8820 (19)	130
C18A—H18A···O3B^ii^	0.95	2.58	3.249 (2)	128
C21A—H21A···F1B^iii^	0.95	2.48	3.2047 (18)	134
C24A—H24C···O3B	0.98	2.48	3.2724 (19)	137
C24A—H24A···Cg1^iv^	0.98	2.50	3.3612 (19)	147

Symmetry codes: (i) −*x*+1, −*y*+1, −*z*+1; (ii) *x*, *y*+1, *z*; (iii) *x*+1, *y*, *z*; (iv) −*x*+2, −*y*+1, −*z*+1.
